# Transcriptomic Validation of the Protective Effects of Aqueous Bark Extract of *Terminalia arjuna* (Roxb.) on Isoproterenol-Induced Cardiac Hypertrophy in Rats

**DOI:** 10.3389/fphar.2019.01443

**Published:** 2019-12-10

**Authors:** Gaurav Kumar, Nikhat Saleem, Santosh Kumar, Subir K. Maulik, Sayeed Ahmad, Manish Sharma, Shyamal K. Goswami

**Affiliations:** ^1^School of Life Sciences, Jawaharlal Nehru University, New Delhi, India; ^2^Department of Pharmacology, All India Institute of Medical Sciences (A.I.I.M.S.), New Delhi, India; ^3^Bioactive Natural Product Laboratory, Department of Pharmacognosy & Phytochemistry, School of Pharmaceutical Education & Research, New Delhi, India; ^4^Peptide and Proteomics Division, Defence Institute of Physiology and Allied Sciences (DIPAS), Defence Research and Development Organisation, New Delhi, India

**Keywords:** *Terminalia arjuna*, cardiac hypertrophy, heart failure, transcriptomics, biological network

## Abstract

Aqueous extract of the bark of *Terminalia arjuna* (TA) is used by a large population in the Indian subcontinent for treating various cardiovascular conditions. Animal experiments have shown its anti-atherogenic, anti-hypertensive, and anti-inflammatory effects. It has several bioactive ingredients with hemodynamic, ROS scavenging, and anti-inflammatory properties. Earlier we have done limited proteomic and transcriptomic analysis to show its efficacy in ameliorating cardiac hypertrophy induced by isoproterenol (ISO) in rats. In the present study we have used high-throughput sequencing of the mRNA from control and treated rat heart to further establish its efficacy. ISO (5 mg/kg/day s.c.) was administered in male adult rats for 14 days to induce cardiac hypertrophy. Standardized aqueous extract TA bark extract was administered orally. Total RNA were isolated from control, ISO, ISO + TA, and TA treated rat hearts and subjected to high throughput sequence analysis. The modulations of the transcript levels were then subjected to bio-informatics analyses using established software. Treatment with ISO downregulated 1,129 genes and upregulated 204 others. Pre-treatment with the TA bark extracts markedly restored that expression pattern with only 97 genes upregulated and 85 genes downregulated. The TA alone group had only 88 upregulated and 26 downregulated genes. The overall profile of expression in ISO + TA and TA alone groups closely matched with the control group. The genes that were modulated included those involved in metabolism, activation of receptors and cell signaling, and cardiovascular and other diseases. Networks associated with those genes included those involved in angiogenesis, extracellular matrix organization, integrin binding, inflammation, drug metabolism, redox metabolism, oxidative phosphorylation, and organization of myofibril. Overlaying of the networks in ISO and ISO_TA group showed that those activated in ISO group were mostly absent in ISO_TA and TA group, suggesting a global effect of the TA extracts. This study for the first time reveals that TA partially or completely restores the gene regulatory network perturbed by ISO treatment in rat heart; signifying its efficacy in checking ISO-induced cardiac hypertrophy.

## Introduction

Pathological cardiac hypertrophy (CH) is a major risk factor for heart failure and mortality worldwide. It is characterized by an increase in length and breadth of the cardiac myocytes in association with other physiological/biochemical changes affecting myocardial blood flow, diastolic function, and diminished cardiac output (Shimizu and Minamino, 2016). Being multifactorial in nature, CH is characterized by fibrosis, pro-inflammatory milieu, autophagy, apoptosis, oxidative stress, altered mechanotransduction, and mitochondrial energetics etc. (Marian and Braunwald, 2017; Nakamura and Sadoshima, 2018). At the molecular level, CH is often associated with an extensive re-programing of gene expression in the heart (Raghow, 2016).Such extensive alterations in cardiac functions is presumably not caused due to the perturbation of a single pathway, but rather a cumulative alteration of many signaling modules including cAMP, PKA, PLC, MAPK, PI3K/Akt, m-TOR etc. (Liu and Molkentin, 2016; Wang and Cai, 2017; Gibb and Hill, 2018; Sciarretta et al., 2018). While these signaling networks are highly interactive and complex, the categorical goal of pharmacological science is to target the nodal points and the effectors (Walters et al., 2016; Philipson et al., 2017).

However, the current treatment option available for CH is limited to few drugs like β-blockers, ACE inhibitors, and angiotensin receptor blockers (Talan et al., 2011; Soliman and Prineas, 2017). Although these drugs are being used worldwide for managing CH, they are also associated with various side effects and their efficacy depends upon several variables including pharmacogenomics (Lainscak et al., 2016; Takezako et al., 2017).

In pursuit of finding novel therapy of CH and heart failure, various natural product based preparations are also empirically used due to the evidences of their efficacy, minimal side effects, affordability, and wide acceptability, especially for the people beyond the western hemisphere (Van Galen, 2014; Rastogi et al., 2016). A number of traditional Chinese medicines like QSYQ and Danshensu (*Salvia miltiorrhiza*) have shown cardio-protective effect in many experimental sets up (Zhu et al., 2016; Hao et al., 2017; Layne and Ferro, 2017).

One major impediment of testing the efficacy of phytochemicals in ameliorating diseases is the difficulty in identifying the active ingredient(s) from a complex mixture of bioactive molecules and their targets of actions. While the first can be addressed by standardization of the extracts used in various assays, the latter issue is more complex and ambiguous. It is now acknowledged that the ingredients of many Ayurvedic preparations, traditional Chinese medicines, and even modern pharmacological agents show synergistic as well as antagonistic effects when added together (Zegpi et al., 2009; Parasuraman et al., 2014; Zhang et al., 2014). Therefore, it is more prudent to test the Ayurvedic formulations as the practitioners use it. However, applications of the modern tools of genomics and proteomics that are broad based, quantitative, and largely unbiased, can help us establish the efficacy of phytomedicines in a more unambiguous manner.

In the Indian subcontinent, the stem barks powder of *Terminalia arjuna* (Roxb.) (TA) has been in use as a cardioprotective agent for centuries by Indian system of medicine (Ayurveda) (Mongalo et al., 2016; Amalraj and Gopi, 2017). Studies done in our laboratory have shown that the bark extract of TA has beneficial effects in experimentally induced myocardial ischemia, hypertrophy, fibrosis, and other cardiovascular disorders. It boosts anti-oxidant activities, prevents fibrosis, protects against ischemia reperfusion injury and has anti-hypotensive effects (Kumar et al., 2009; Maulik and Katiyar, 2010; Maulik et al., 2016; Meghwani et al., 2017). In a recent study, arjunolic acid, one of the constituents of the aqueous TA extract, was shown to ameliorate cardiac fibrosis by inhibiting TGF-β signaling (Bansal et al., 2017). Earlier, we had used limited proteomic approach (2D gel based) to establish that TA bark extract substantially modulates the rat cardiac proteome under adrenergic (ISO) stress (Kumar et al., 2017). To further establish the efficacy of TA extract we now have used more robust approach that is global transcriptomic analyses to establish the efficacy of TA extract in modulating various biological pathways and gene networks targeted by ISO. We demonstrate that TA extract reverses ISO induced reprogramming of gene expression in rat heart. Our study for the first time convincingly establishes that the effects of TA are far wider than that is expected for a modern drug usually having a single target.

## Materials and Methods

### Animal Experiments and Ethics Statement

Laboratory bred Wistar male rats (150–200 g, 10–12 weeks) were employed for the study and maintained under standard laboratory conditions (temperature; 25°C ± 2°C, relative humidity; 50% ± 15% and 12-h dark/12-h light period). The study was conducted in accordance with the Institutional Animal Ethics Committee, All India Institute of Medical Sciences, New Delhi, India. All animal care and experimental protocols were performed in compliance with the National Institutes of Health (NIH) Guidelines for the Care and Use of Laboratory Animals (NIH Publication no. 85723, revised 1996).

### TA Extract

The material under investigation is a standardized aqueous extract of the bark of TA (made available to us from the Dabur Research Foundation, Ghaziabad, India). It has been is prepared by extraction as per the literatures of Ayurveda against its traditional claims and as it has been extensively used in the Indian System of Medicine for centuries. For its scientific validation, the extract has been standardized by the fingerprinting using UPLC. All the separated metabolites of the extract have also been characterized by metabolomic profiling using UPLC high resolution mass spectrometry which separated 26 compounds at different Rt with different molecular masses (**Supplemental Figures S1A, B**). The major peaks obtained were at Rt 8.01, 7.80, and at 7.53 with m/z 510.26, 893.46, and 488.25 respectively (**Supplemental Figure S1C**). The major peak at Rt 7.80 was identified as Arjunolic acid with m/z 488.25 (**Supplemental Figure S1D**) which is main marker compound of Arjuna (Kumar et al., 2017).

### Administration of TA Extract

The animals were randomly divided into four groups according to drug treatment with5 animals in each group. The groups consisted of control, ISO + saline, ISO_TA, and TA alone. In control group the animals were administered normal saline, 1.0 ml/kg body weight, s.c. once daily for 14 days. In ISO + saline group, rats received both ISO, 5.0 mg/kg body weight s.c. once daily followed by normal saline, 1 ml/kg body weight, orally once daily for 14 days. In the group designated with ISO_TA, rats were administered with ISO, 5.0 mg/kg body weight, s.c. once daily along with the aqueous extract of TA, 125.0 mg/kg body weight, orally once daily for 14 days. In TA group, aqueous extract of TA, 125.0 mg/kg body weight, was administered orally once daily for 14 days. Lyophilized powder of aqueous extract of TA stem bark and ISO was freshly prepared in double distilled water before use.

### RNA Isolation

The rats were sacrificed at the end of treatment and the hearts were carefully excised followed by snap freezing at −80°C. The tissues samples from five animals were pooled for each group and total RNA was isolated utilizing TRI Reagent (Sigma Aldrich) as per manufacturer’s instructions. The integrity of RNA was checked on 0.8% formaldehyde agarose gel and further purified for gene expression experiments employing RNeasy Mini Kit (Qiagen, Hilden, Germany) with on-column DNAase digestion.

### RNA Sequencing

#### Library Preparation and Sequencing

The respective cDNA libraries were prepared using TruSeq mRNA Sample Prep Kit (Illumina, Inc), as per manufacturer’s instructions. Poly-A tail containing mRNA molecules were purified using oligo-dT attached magnetic beads (Illumina, Inc) using two rounds of purification. Purified mRNA were subjected to fragmentation, reverse transcription, end repair, 3′- end adenylation, and adaptor ligation, followed by PCR amplification and bead purification. The unique barcode sequences were incorporated in the adaptors for multiplexed high-throughput sequencing. The final product was assessed for its distribution of size and concentration using 2100 Bioanalyzer (Agilent Technologies), followed by cluster generation and sequencing on HiSeq 2000.

#### Read Filtering, Processing, and Alignment

The mRNA-Seq reads were aligned to the rat reference genome (downloaded from UCSC) utilizing “Bowtie 2” and “TopHat” as per published method (Langmead and Salzberg, 2012; Ghosh and Chan, 2016). The mapped reads were subsequently fed as input to “Cufflinks” (Langmead and Salzberg, 2012). The assembly files so generated were merged into a unified annotation with the reference transcriptome annotation for further analysis. The merged annotations were quantified using “Cuffdiff” to identify differentially expressed genes (Langmead and Salzberg, 2012). The “FPKM” (fragments per kilobase of transcript per million fragments) parameter was used to quantitate abundance of the transcripts (Li et al., 2017).

### Bioinformatics Analysis

Gene Ontology, Pathway Mining, Functional Annotation Clustering was done utilizing various data mining tools. David Bioinformatics resources, which use a previously published gene, list to statistically highlight the most overrepresented (enriched) biological annotation viz., Gene Ontology Terms (Huang et al., 2007; Xing et al., 2016). GeneMANIA, a Cytoscape plug-in were further used for data analysis (Montojo et al., 2010).GeneMANIA, was used to place the data obtained in a functional context and represent it as degree sorted circular view of key networks of statistically significant biological processes. The GeneMANIA integrates association networks from multiple sources into a single composite network using a conjugate gradient optimization algorithm.

## Results

### Treatment With TA Extracts Blunts the Activated β-Adrenergic Signaling in Heart

In order to understand the molecular mechanisms of cardioprotection by the TA extracts, we analyzed its effects on the overall signatures of β-adrenergic stimulation in rat heart. Rats were injected with ISO (5 mg/kg, once daily, s.c.) and fed with the aqueous TA extract (125 mg/kg, once daily, orally). After 28 days of treatment, the animals were sacrificed and the effects of TA treatment were confirmed at the gross morphological level by weighing the hearts. In reiteration of our previous reports (Kumar et al, 2017), while ISO treatment resulted in an increase in heart to body weight ratio, TA extracts reversed it significantly. As expected, TA alone had no effect on heart to body weight ratio (**Supplemental Figure S2**).

**Figure 1:**
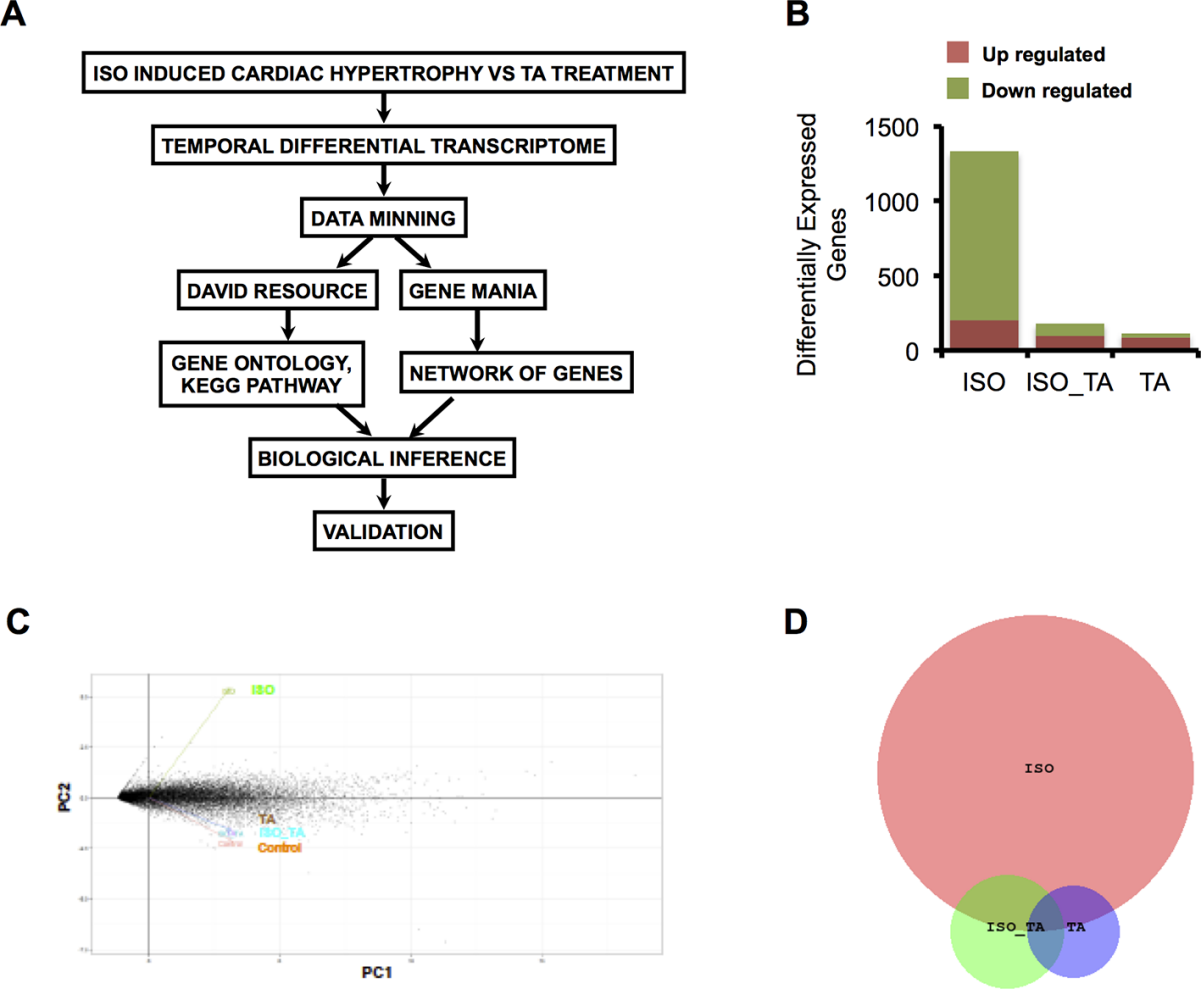
**(A)** Scheme of data mining workflow for analyzing the transcriptome data. **(B)** The bar graph indicates the number of upregulated and downregulated gene in each condition. **(C)** Principal component analysis (PCA) of gene expression data sets from all three groups represented as two-dimensional plot of PC1 (Component 1) and PC2 (Component 2). **(D)** Venn Diagram representing overlap of differentially expressed Genes in ISO, ISO_TA, and TA groups. (Groups: ISO = ISO treated, ISO_TA = ISO + *T. arjuna* treated, TA, *T. arjuna* treated).

To determine molecular basis of the effects of TA on ISO treatment, we performed RNA sequence based differential gene expression analyses. This is a state of the art technology with tremendous potential for revealing multiple molecular cues associated with diseases and their amelioration by the drugs (Doostparast Torshizi and Wang, 2018). A schematic presentation of the experiment pipeline is shown in **Figure 1A**.

The overall number of differentially expressed genes in all the groups is represented as a bar plot in **Figure 1B** and differentially expressed genes in each group along with log2 fold change are tabulated (**Supplemental Tables S1**, **S2**, and **S3**). As expected, treatment with ISO generated a wide response with 1,333 genes differentially expressed of which 1,129 were downregulated and 204 were upregulated. In striking contrast, pretreatment of animals with the aqueous TA bark extracts led to a marked reduction of this expression signature, with only 97 genes upregulated and 85 genes downregulated. The TA alone group consisted of 88 upregulated and 26 downregulated genes. Notably, when we subjected the genes modulated by all the groups in principal component analysis, we observed ISO_TA and TA alone groups clustered closely with control group while the ISO group was distinctively separated from this cluster (**Figure 1C**). This analyses showing higher similarity between control, ISO_TA, and TA alone groups thus reiterates our early report that TA suppresses the responses induced by ISO treatment (Kumar et al, 2017). Finally, the intersection of differentially expressed genes between ISO, ISO_TA, and TA group is shown in **Figure 1D**. It is evident from the data that a large number of genes which were induced in ISO group, are missing from the ISO_TA group. Taken together, these results indicate that there are unique gene expression signatures for all groups of animals treated with ISO, TA, or both.

### TA Treatment Reverses the Metabolic Reprogramming Induced by ISO Treatment

**Figure 2:**
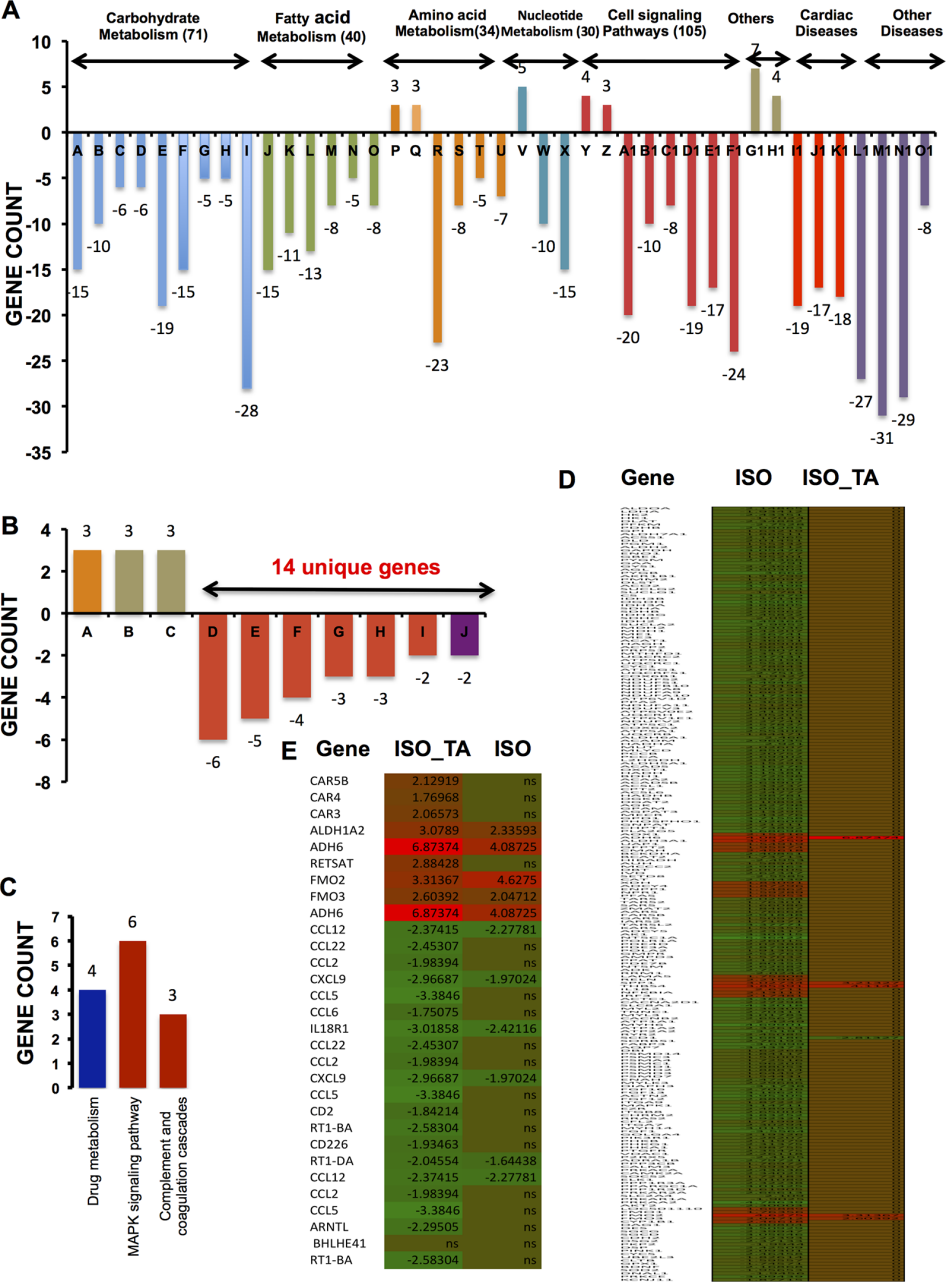
KEGG pathways significantly enriched after multiple testing adjustments (*p* < 0.1) **(A)** Bar graph indicates KEGG pathway upregulated and downregulated after ISO treatment. Pathways under the same group (carbohydrate metabolism, fatty acid metabolism, etc.) are clustered together and marked by the same color. The number of unique genes modulated in each group are shown in parenthesis next to the respective group names. The total number of genes modulated in various pathways under each group is generally larger than the total number of unique genes mentioned in parenthesis. It is due to the occurrence of same genes in multiple pathways and accordingly, those were counted more than once. The individual pathways given in the bar graph (A-O1) is tabulated in **Table 1**. **(B)** Bar graph representing genes upregulated and downregulated by ISO + TA is shown. The total number of unique genes that were downregulated is 14. The total number of genes modulated in various pathways under each group is more than 14 as the same genes might be associated with multiple pathways, hence counted more than once. **(C)** Bar graph representing three different pathways involving 13 unique genes upregulated by TA alone is shown. The individual pathways given in the bar graphs in A and B is tabulated in **Table 1**. **(D)** Heatmap of significantly regulated genes in the ISO group compared to ISO_TA group along with expression value. **(E)** Heatmap of significantly regulated genes in the ISO _TA group compared to ISO group along with expression value. The genes, which are not significantly regulated, are indicated as ns (non-significant). A detail of expression value of heatmap along with p-value is tabulated in **Supplementary Table S5**.

To elucidate a discernible reversal of ISO-induced differential gene expression pattern by TA, we analyzed the repertoire of regulated genes in different treatment groups using various data mining strategies. We first used “DAVID bioinformatics resources” for systematic and integrative analysis of large data set and extracted key biological pathways (KEGG) that were significantly (*p* < 0.1) regulated. As shown in **Figure 2A** and **Table 1**, the pathways that are downregulated in ISO treated group includes those involved in the metabolism of carbohydrates (71 genes), fatty acids (40 genes), amino acids (34 genes), and nucleic acids (30 genes); receptor activation and cell signaling (105 genes); and cardiovascular and other diseases (160 genes). As shown in **Figure 2A**, among the 71 unique genes involved in carbohydrate metabolism were those associated with glycolysis and gluconeogenesis (Bar A: 15 genes); metabolism of starch and sucrose (Bar B: 10 genes), fructose and mannose (Bar C: six genes), and galactose (Bar D: six genes); TCA cycle (Bar E: 19 genes), pyruvate metabolism (Bar F: 15 genes); pentose phosphate pathway (Bar G: five genes); metabolism of mannose, glyoxylate, and dicarboxylate (Bar H: five genes); and oxidative phosphorylation (Bar I: 28 genes). Similarly, 40 downregulated genes involved in fatty acid metabolism included those associated with the metabolism of propanoate (Bar J: 15 genes) and butanoate (Bar K: 11 genes); fatty acid (Bar L: 13 genes), glycerolipid (Bar M: eight genes); fatty acid elongation in mitochondria (Bar N: five genes); and glycerophospholipid metabolism (Bar O: eight genes). Noticeably, three genes involved in tyrosine metabolism (Bar P) and three genes involved in amino sugar and nucleotide metabolism (Bar Q) were upregulated under ISO treatment. Genes downregulated by ISO were also involved in the anabolism of valine, leucine, isoleucine, and lysine (Bar R: 23 genes); lysine degradation (Bar S: eight genes); and metabolism of beta-alanine (Bar T: five genes) and tryptophan (Bar U: seven genes). Five genes involved in purine metabolism were upregulated under ISO treatment (Bar V). Ten genes involved in aminoacyl-tRNA biosynthesis (Bar W) and seven genes in purine metabolism (Bar X) were also downregulated under ISO treatment. Out of seven genes involved in receptor activation and cell signaling upregulated by ISO, four genes were involved in ECM-receptor interaction (Bar Y) and three in cytosolic DNA-sensing pathway (Bar Z). The largest cluster of genes downregulated by ISO also included those involved in cell signaling and receptor activation. Among the downregulated genes included those involved in cardiac muscle contraction (Bar A1: 20 genes), PPAR signaling (Bar B1: 10 genes), proteostasis (Bar C1: eight genes), regulation of actin cytoskeleton (Bar D1: 19 genes), calcium signaling (Bar E1: 17 genes), and insulin signaling (Bar F1: 24 genes). Out of 160 genes involved in cardiovascular and other diseases modulated by ISO, seven genes involved in drug metabolism (Bar G1) and four genes involved in metabolism of xenobiotics by cytochrome P450 (Bar H1) were upregulated. Among the genes downregulated by ISO, were those associated with hypertrophic, dilated, and arrhythmogenic right ventricular cardiomyopathy (Bar I1: 19 genes, Bar J1: 17 genes, and Bar K1: 24 genes); Huntington’s (Bar M1: 31 genes), Parkinson’s (Bar N1: 27 genes), and Alzheimer’s diseases (Bar O1: 29 genes); type II diabetes (Bar P1: eight genes).

**Table 1:** List of pathways extracted from “DAVID bioinformatics resources.” Codes as indicated in Figure 2 and respective gene counts are shown. Negative and positive sign indicates downregulation and upregulation respectively.

FIGURE 2A					
Code	KEGG Pathways	Gene count	Code	KEGG pathways	Gene count
	**Carbohydrate metabolism**				
A	Glycolysis/gluconeogenesis	-15	B1	PPAR signaling pathway	-10
B	Starch and sucrose metabolism	-10	C1	Proteasome	-8
C	Fructose and mannose metabolism	-6	D1	Regulation of actin cytoskeleton	-19
D	Galactose metabolism	-6	E1	Calcium signaling pathway	-17
E	Citrate cycle (TCA cycle)	-19	F1	Insulin signaling pathway	-24
F	Pyruvate metabolism	-15		**Others**	
G	Pentose phosphate pathway	-5	G1	Drug metabolism	7
H	Glyoxylate and dicarboxylate metabolism	-5	H1	Metabolism of xenobiotics by cytochrome P450	4
I	Oxidative phosphorylation	-28		**Cardiac disease**	
	**Fatty acid metabolism**		I1	Dilated cardiomyopathy	-19
J	Propanoate metabolism	-15	J1	Arrhythmogenic right ventricular cardiomyopathy (ARVC)	-17
K	Butanoate metabolism	-11	K1	Hypertrophic cardiomyopathy (HCM)	-18
L	Fatty acid metabolism	-13		**Other disease**	-27
M	Glycerolipid metabolism	-8	L1	Parkinson’s disease	
N	Fatty acid elongation in mitochondria	-5	M1	Huntington’s disease	-31
O	Glycerophospholipid metabolism	-8	N1	Alzheimer’s disease	-29
	**Amino acid metabolism**		O1	Type II diabetes mellitus 8	-8
P	Tyrosine metabolism	3			
Q	Amino sugar and nucleotide sugar metabolism	3		**FIGURE 2B**	**Gene Count**
R	Valine, leucine and isoleucine degradation	-23	A	Nitrogen metabolism	3
S	Lysine degradation	-8	B	Retinol metabolism	3
T	beta-Alanine metabolism	-5	C	Drug metabolism	3
U	Tryptophan metabolism	-7	D	Chemokine signaling pathway	-6
				Cytokine-cytokine receptor	
	**Nucleotide metabolism**		E	interaction	-5
V	Purine metabolism	5	F	Cell adhesion molecules (CAMs)	-4
W	Aminoacyl-tRNA biosynthesis	-10	G	NOD-like receptor signalling pathway	-3
X	Purine metabolism	-15	H	Antigen processing and presentation	-3
	**Cell signaling pathway**		I	Circadian rhythm	-2
Y	ECM-receptor interaction	4	J	Asthma	-2
Z	Cytosolic DNA-sensing pathway	3			
A1	Cardiac muscle contraction	-20			

As shown in **Figure 2B**, total of nine genes were upregulated by ISO_TA of which three each were involved in nitrogen, retinol, and drug metabolism respectively (Bar A, B, and C). Total fourteen unique genes were downregulated by the ISO_TA group. Those included genes involved in chemokine and cytokine signaling (Bar D: six genes), cytokine-cytokine receptor interaction (Bar E: five genes), cell adhesion (Bar F: seven genes), NOD-like receptor signaling pathway (Bar G: three genes), antigen processing and presentation (Bar H: three genes), circadian rhythm (Bar I: two genes), and the development of asthma (Bar J: two genes).

Only thirteen genes were upregulated by TA alone as shown in **Figure 2C**. Those were involved in drug metabolism (Bar A: four genes), MAPK signaling (Bar B: six genes), and complement cascade (Bar C: three genes). Taken together, total 316 unique genes were downregulated by ISO, and their levels were restored upon TA treatment (**Figures 2D, E**). This suggests a genome wide effect of TA extract in reversing the pathological effects of ISO.

### TA Treatment Ameliorates the Pathological Networks Activated by the Adrenergic Stimulation

**Figure 3:**
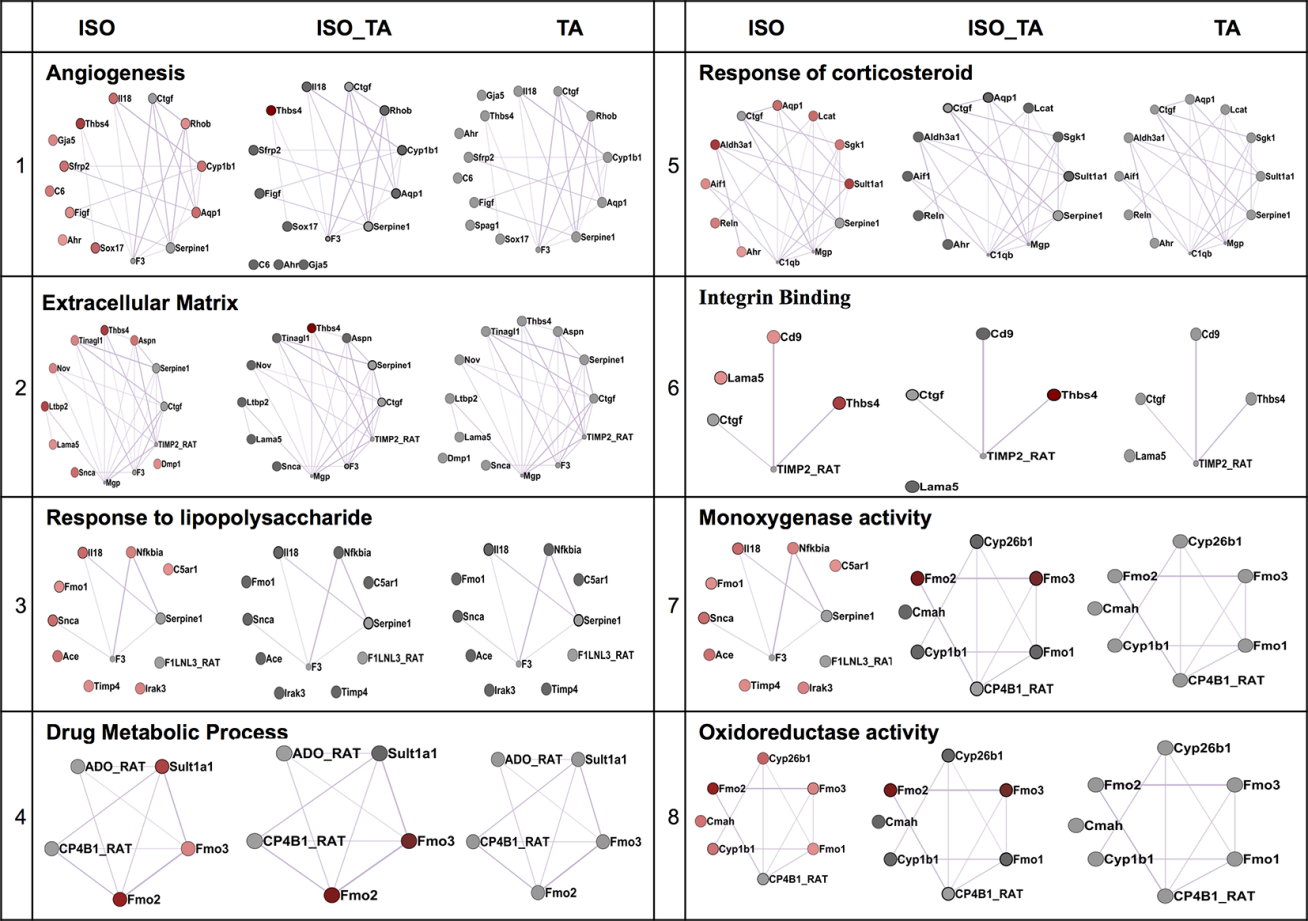
Comparison of enriched non-redundant biological processes represented as “degree sorted circular view” networks of upregulated genes in ISO group extracted from “GeneMANIA” (Cytoscape plugins) along with ISO_TA and TA group overlaid network. Upregulated, downregulated, and undifferentiated gene represented as red node, green node, and gray node respectively.

**Figure 4:**
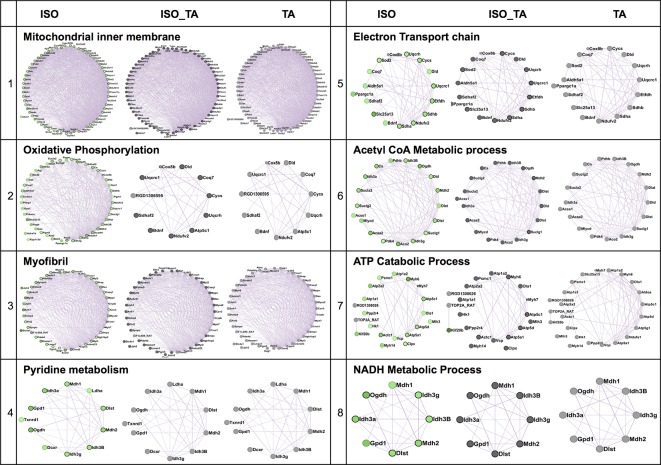
Comparison of enriched non-redundant biological processes represented as “degree sorted circular view” networks of downregulated genes in ISO group extracted from "GeneMANIA" (Cytoscape plugins) along with ISO_TA and TA group overlaid network. Upregulated, downregulated, and undifferentiated gene represented as red node, green node, and gray node respectively.

We next applied a widely used network extraction tool GeneMANIA to mine various gene networks and associated functions from these data sets (Montojo et al., 2010). Based on the functional similarity or shared topology in the published literature, this tool exhibit networks from the query gene list. The network thus derived were then visualized and analyzed using the visualization software Cytoscape 3.0.2. We derived networks of up- and downregulated genes in all the treatment sets as shown in **Figures 3** and**4** where the extent of modulation are represented by color codes as red: upregulation; green: downregulation; and gray: no significant change. These networks indicate modulation of various key genes, reflecting the diversity of ISO-induced reprogramming of cellular networks and its restoration by TA. In agreement with the data obtained from KEGG pathways, this analysis showed the upregulation of biological networks associated with angiogenesis, extracellular matrix organization, integrin binding, inflammation, drug metabolism, redox metabolism, and corticosteroid response in the ISO-treated group (**Figure 3**). ISO also downregulated the networks like those involved in oxidative phosphorylation, pyrimidine metabolism, myofibril, mitochondrial electron transport chain, and acetyl CoA metabolism (**Figure 4**). The details of the network along with key genes upregulated and downregulated by ISO is given in **Table 2**.

**Table 2:** List of networks extracted from GeneMANIA after ISO treatments along with key genes are indicated.

*Network upregulated by ISO treatment*		
S.no	Network	Key gene
	Angiogenesis	c-fos induced growth factor (Figf)
A		Vascular endothelial growth factor D (Vegf D)
		Connective tissue growth factor (Ctgf)
		Gap junction protein alpha 5 (Gja5)
		Thrombospondin 4 (Thbs4)
B	Extracellular matrix	Laminin subunit alpha 5 (Lma5)
		Latent transforming growth factor beta binding protein 2 (Ltbp2)
	Integrin binding	Metalloproteinase inhibitor 2 (Timp2 )
C		Cd9 antigen (Cd9)
	Inflammation	Interleukin-18 (Il18)
D		NFKB inhibitor alpha (Nfkbia)
		Interleukin 1 receptor associated kinase 3 (IRAK3)
	Drug metabolism	Cytochrome P450 (Cyp1b1)
E		Flavin containing monooxygenase 3 (Fmo3)
		TIMP metallopeptidase inhibitor 4 (Timp4)
	Redox metabolism	Sulfotransferase family 1A member 1 (Sulta1)
F		Aminoethanethiol dioxygenase (Ado)
	Corticosteroid response	Serum/glucocorticoid regulated kinase 1 (Sgk1)
G		Aldehyde dehydrogenase 3 family member A1 (Aldh3a1)
***Network downregulated by ISO***		
H	Oxidative phosphorylation	ATP synthase (Atp5a1),
	Translocase of inner mitochondrial membrane 44 homolog	
		(Timm4)
		Mitochondrial carrier 2 (Mtch2)
		Malate dehydrogenase 2 (Mdh2)
		Ubiquinol-cytochrome c reductase hinge protein (Uqcrh)
		NADH dehydrogenase subunit (NdufV2)
	Pyrimidine metabolism	Oxoglutarate (alpha-ketoglutarate) dehydrogenase (Ogdh)
I		Thioredoxin reductase 1 (Txnrd1)
		Glyceraldehyde-3-phosphate dehydrogenase,(Gpd1)
	Myofibril	Ankyrin repeat domain 23 (Ankrd2)
J		Intracellular hyaluronan binding protein4 (Habp4)
		Junctophilin 1 (Jph1)
	Mitochondrial electron transport	Succinate dehydrogenase flavoprotein subunit (Sdha1)
K	Flavoprotein dehydrogenase (Etfdh)	
	Acetyl CoA metabolism	Acetyl-CoA acyltransferase 1 (Mdh1)
L		NAD-dependent glycerol-3-phosphate dehydrogenase (Gpd1)

To characterize the impact of pretreatment with TA extracts (ISO_TA) on the metabolic reprogramming elicited by ISO, we overlaid the genes regulated in this groups on the network derived from ISO groups. As shown in **Figures 3** and**4**, nodes in the overlaid network in ISO_TA and TA group showed largely no significantly regulated genes (gray color). Clearly, the metabolic network activated in ISO group was completely absent in ISO_TA (and TA group), suggesting a global effect of the TA extracts. Moreover, TA treatment alone significantly perturbed only a few networks as a limited number of genes were modulated by TA-treatment (**Figures 3** and **4**).

## Discussion

Despite the advancements and availability of modern medicines, especially in past quarter of a century, a large population worldwide still relies upon the traditional medicines (Van Galen, 2014). Although traditional medicines are used worldwide, it is prevalent in the Asian subcontinent, especially in India and China (Jaiswal et al., 2016). Due to sustained support from the government, traditional medicines in China are better regulated and have more acceptability among its own population. Several ancient Chinese formulations have also been successfully exploited for developing modern drugs that are now widely used by the western community (Chao et al., 2017).

Under the ambit of clinical bioinformatics, high-resolution, high-throughput data analyses has enhanced opportunities for examining the efficacies of traditional medicinal preparations. Applications of sophisticated tools of microarray, next generation sequencing, pharmacogenomics etc. enhances our understanding of the mode of actions of various formulations. These advance tools of analytical biology thus provide an opportunity to validate or refute the therapeutic potential of phytomedicines (Beckmann and Lew, 2016). Although the TA extract is widely used for treating various types of cardiovascular ailments by traditional practitioners in the Indian subcontinent, any systematic study of its efficacy using the modern tools of biology has not been done till date (Maulik and Talwar, 2012).

ISO, an adrenergic agonist, is a widely used for studying cardiac pathobiology in experimental animals (Seifert, 2013). However, studies on its effects on gene expression in the heart have largely been restricted to a handful of genes of interest and fewer analyses of the total transcriptome from ISO treated heart have been reported till date (Li et al., 2003; Lu et al., 2012; Talarico et al., 2014). To establish the efficacy of TA in ameliorating CH, we undertook the total transcriptomic analyses of ISO/TA treated rat heart. Surprisingly, we found that total 1129 genes were downregulated and 204 genes were upregulated by the ISO treatment and most of them were restored by TA. Since adrenergic receptors play a critical role in cardiac functions, modulation of such a large number of genes upon sustained ISO treatment might not be unusual. It is likely that the ISO treated heart needs to reset its function through the readjustment of its genome wide expression profile. To be noted that Talarico et al. (2014) have reported change in expression of 493 genes in mouse heart following ISO treatment for an hour. Considering the species difference and experimental parameters like the dose and duration of treatment, our data is largely in tune with theirs (**Supplementary Table S4**).

In our study, among the 1,333 genes that were modulated by ISO (**Table 2**, **Supplemental Data**) included those involved in metabolism (carbohydrate, fatty acid, and nitrogen; total 105 genes), cell signaling (105 genes), and cardiovascular and other diseases (106 genes). Changes in metabolism such as a shift from oxidative phosphorylation to glycolysis and increased fatty acid oxidation are the hallmarks of heart failure (Fillmore et al., 2014; Fukushima et al., 2015). Since intermediary metabolism involves substantial number of enzymes, modulation of a large number of metabolic genes by ISO is expected, although its significance needs further study. Similarly, adrenergic signaling plays a major role in cardiovascular function and diseases. The mechanisms of adrenoceptor activation, its downstream targets, its signal specificity versus bias and receptor dynamics have been extensively studied for understanding the role of adrenergic system in cardiovascular pathophysiology (Lohse, 2015). Also, the cross talk between adrenergic signaling and that by other receptor viz., insulin, EGF and TGFβ play a critical role in cardiovascular function (Saha et al., 2012; Heger et al., 2016; Fu et al., 2017; Mohan et al., 2017). Signaling by these receptors in heart involve a plethora of kinases, phosphatases, second messengers and gene regulatory proteins. Therefore, changes in expression of more than hundred signaling genes by the sustained adrenergic activation is quite likely.

Although an exhaustive analyses of the role of these diverse group of genes in cardiovascular biology would be desired, it might as well lead to a voluminous but hypothetical compilation of experimental data accumulated over the past several decades. Therefore, we intend to focus onto the relevance of 54 genes that are modulated by ISO treatment and are involved in cardiac diseases. It was quite convincing to find that number of genes downregulated by ISO are involved in Redox-homeostasis/oxidative stress and noted among them is glutathione-S-transferase (GST, **Table 3**). Glutathione-S-transferase family are Phase II detoxification enzymes that catalyze the covalent conjugation of glutathione (GSH) to electrophilic compounds such as peroxidized lipids, enabling their breakdown. Under oxidative stress, various enzymatic and other proteins susceptible to oxidative damage also undergo S-glutathionylation, a process of covalent conjugation of glutathione to cysteine sulfhydryl or sulfenic acid groups. In a recent study, a member of the GST family, i.e. GSTπ has been shown to catalyze protein S-glutathionylation *in vivo* (Townsend et al., 2009).Downregulation of a number of redox enzymes including GST thus suggest a reduced capacity of the heart to cope up with increased oxidative stress under ISO treatment (Saleem et al., 2018).

**Table 3:** Functional class of cardiac disease related genes modulated after ISO treatment.

S.no	Functional class	Gene name
1	Redox homeostasis	1. Glutathione S-transferase A6 (LOC501110) 2. Dimethylaniline monooxygenase (Fmo1, Fmo2, Fmo3) 3. Aldehyde oxidase 1 (Aox1) 4. Alcohol dehydrogenase 6 (Adh6) 5. Aldehyde dehydrogenase (Aldh3a1),
2	Sarcomere function	1. Myosin light chain, phosphorylatable (Mylpf) 2. Myosin regulatory light chain 2 (Mlc2) 3. Myosin light chain kinase 3 (Mylk3) 4. Myosin light chain 3 (Myl3) 5. Troponin C1 (Tnnc1) 6. Myosin-6; Muscle contraction (Myh6) 7. Actinin alpha 2 (Actn2) 8. Actin, alpha cardiac muscle 1 (Acta2) 9. Myosin light chain kinase 3 (Mylk3)
3	Cell/Ca^++^ signaling	1. 5′-AMP-activated protein kinase catalytic subunit alpha-2 (Prkaa2) 2. Protein phosphatase 1E (Ppm1e) 3. Protein phosphatase 1B (Ppm1b) 4. Serine/threonine-protein kinase (STK11) 5. Sarcoplasmic/endoplasmic reticulum calcium ATPase 2 (Atp2a2) 6. Matrix metalloproteinase-24 (Mmp24) 7. Cadherin-2 (Cdh2) 8. 3-phosphoinositide-dependent protein kinase 1 (Pdpk1) 9. Calsequestrin-2 (Casq2) 10. Ryanodine receptor 2 (Ryr2)

Another noticeable observation was the downregulation of expression of a number of contractile proteins viz., Troponin C1, β-Myosin heavy chain, Myosin light chain 2 etc. (**Table 3**). Modulation of expression of contractile proteins have been demonstrated in *ex vivo* cultured myocytes and in experimental rats treated with adrenergic agonists. While in *ex vivo* rat cardiac myocytes, the level of myosin light chain 2 mRNA increases upon treatment with phenylephrine; the mRNA for α-myosin heavy chain decreases in aged spontaneously hypertensive rats with heart failure (Shubeita et al., 1992; Boluyt et al., 1994). In male Sprague-Dawley rats, infusion of NEfor seven days reduce the expression of myosin heavy chain 11, myosin light chain 3 and troponin I. In patients with heart failure, the expression of a number of sarcomeric genes decreases, while their expression is restored in those having left ventricular assisted device (Rodrigue-Way et al., 2005). Taken together, our observation that ISO treatment reduces the mRNA levels of certain sarcomeric genes are in general agreement with studies done by others and the restoration of their levels by TA treatment is significant.

Another set of genes whose expression were downregulated by ISO are involved in cell signaling in general and Ca^++^ signaling in particular. As summarized in **Table 3**, ISO treatment reduced the expression of Ryanodine receptor, Calsequestrin-2, Sarcoplasmic/endoplasmic reticulum calcium ATPase 2 and cardiac phospholamban. Except that of calsequestrin-2, the expression of others decrease in various experimental models of CH and heart failure and in human patients (Nagai et al., 1989; Takahashi et al., 1992; Arai et al., 1996; Kawase and Hajjar, 2008). The mRNA levels of three other genes encoding signaling kinases viz., adenylate cyclase, PKA, and SSTK11 were also downregulated by ISO and restored by TA. The role of adenylate cyclase-PKA in CH has been extensively studied, especially in the context of adrenergic signaling. Available literature suggests that the compartmentalization of cAMP/protein kinase A (PKA) in different subcellular organelles is involved in pathophysiological outcomes under different stimuli (Fields et al., 2016). However, upon extensive literature search, we could not find any report on modulation of expression of these two key enzymes under any pathological context, hence we are unable to correlate our data with that of others. The downregulation of the third kinase, i.e. SSTK11 (Liver Kinase B1/LKB1) in ISO treated heart and its restoration by TA is notable as it prevents CH and dysfunction (Ikeda et al., 2009).

Taken together, above mentioned alteration in the profile of expression of genes involved in metabolism, signaling, and pathology of heart failure are well in conformity of available literature. Further, although substantial literature has shown the beneficial effects of TA in ameliorating various cardiovascular dysfunction, to date no information is available on the modulation of gene expression by TA. Therefore our observation that TA restores a vast majority of genes upregulated and downregulated by ISO in heart is of immense importance. However, TA extract is a mixture of about 26 constituents of which several have been shown to be bioactive (Saha et al, 2012; Kumar et al., 2017). Also, the effects of TA is pleiotropic, as it has anti-atherogenic, hypotensive, inotropic, anti-inflammatory, anti-thrombotic, and antioxidant actions (Maulik and Talwar, 2012). Till date, very little is known about the mechanism of actions of TA at the cellular and molecular levels. Thus, at this point we are unable to offer the mechanistic insight into the global reversal of the effect of ISO by TA. Nevertheless, our present study thus opens up a new window of understanding the beneficial role of TA extract in treating cardiovascular ailments.

## Data Availability Statement

The RNA seq data generated in this study have been submitted to the NCBI BioProject database (https://www.ncbi.nlm.nih.gov/bioproject/) under accession number PRJNA525742.

## Ethics Statement

The study was conducted in accordance with the Institutional Animal Ethics Committee, All India Institute of Medical Sciences (AIIMS), New Delhi, India. All animal care and experimental protocols were performed in compliance with the National Institutes of Health (NIH) guidelines for the care and use of the Laboratory Animals (NIH Publication no. 85723, revised 1996).

## Author Contributions

GK has done the data analyses and MS has supervised him. NS has done the animal experiments while SK had done the experimental work at the very early phase. SKM had conceived the entire project. SA had helped in analyzing the TA extract. MS and SKG wrote the manuscript.

## Funding

The authors thankfully acknowledge funding support from the DST-PURSE awarded by the Department of Science and Technology, Government of India to the Jawaharlal Nehru University. Also, the initial work was done with the support from the Department of Science and Technology under the grant SR/SO/HS-0085/2012 awarded to SKG. GK is a recipient of a NPDF from SERB-DST, Government of India.

## Conflict of Interest

The authors declare that the research was conducted in the absence of any commercial or financial relationships that could be construed as a potential conflict of interest.
