# Supplementary material for: Transcriptomic Validation of the Protective Effects of Aqueous Bark Extract of Terminalia arjuna (Roxb.) on Isoproterenol-Induced Cardiac Hypertrophy in Rats
Source: Front Pharmacol. 2019 Dec 10;10:1443. doi: 10.3389/fphar.2019.01443 (PMC6916006; doi:10.3389/fphar.2019.01443)
Supplement: Supplementary file 1 [file DataSheet_1.docx]

**SUPPLEMENTARY FIGURES LEGENDS**

**Supplementary Figure 1S** Ultra-Performance Liquid Chromatography-Mass Spectrometry (UPLC-MS). Water’s ACQUITY UPLC(TM) system (Serial No # F09 UPB 920M; Model code # UPB, Waters Corp., MA, USA) equipped with a binary solvent delivery system, an auto-sampler, column manager and a tunable MS detector (Serial No # JAA 272; Synapt; Waters, Manchester, UK) installed and controlled by Mass Lynx V 4.1 (Waters, USA) with C18 column (ACQUITY UPLC(R) BEH C18, 1.7 µm, 2.1 x 100 mm) was used for analysis. The chromatography of extract in methanol (500 µg/mL) was performed on solvent acetonitrile (A) and 0.5% v/v formic acid in water (B) in which separation was achieved on gradients elution mode (Initially, 10% A, 90%B; 0-17 min 90% A, 10%B; 17-19 min 10% A, 90%B). The run time was 20 min with flow rate of 0.5 mL/min. Flow rate of nebulizer gas was set to 10 µL/min, for cone gas, it was set to 50 L/h and the source temperature was fixed to 100 ºC. The capillary and cone voltages were set to 3.0 and 40 KV, respectively. For collision, argon was employed at a pressure of 5.3 х 10-5 Torr. The accurate mass and composition for the precursor ions and for the fragment ions were calculated using the Mass Lynx V 4.1 software incorporated in the instrument.

**Supplementary Figure 2S:**  TA extract restores heart weight by body weight ratio in ISO–induced cardiac hypertrophy. Rat groups (n=5) were administered with either ISO (5 mg/kg/day) alone or aq. TA (125mg.kg/day, pre-administered) followed by ISO for 14 days. Control rats were administered with saline (1ml/kg/day) only. Post-mortem measurements of HW/BW (mg/gm x10-3) were done to assess cardiac hypertrophy

**SUPPLEMENTARY TABLE LEGENDS**

**Supplementary Table 1S:** List of differentially expressed genes (compared to Controls), as inferred from of RNA seq analysis after ISO treatment. Gene Names and respective Fold change (Log_2_) are shown.

**Supplementary Table 2S:** List of differentially expressed genes (compared to Controls), as inferred from of RNA seq analysis after ISO +TA treatment. Gene Names and respective Fold change (Log_2_) are shown.

**Supplementary Table 3S:** List of differentially expressed genes (compared to Controls), as inferred from of RNA seq analysis after TA treatment. Gene Names and respective Fold change (Log_2_) are shown.

**Supplementary Table 4S:** Comparative gene expression value in Fold change (Log2) post ISO treatment in current experiment and Talarico et al Plos one data.

**Supplementary Table 5S:** Comparative gene expression value in Fold change (Log2) along with P-value of genes shown in heatmap in figure 2D and 2E.
