# Supplementary figures and images for: Transcriptomic Validation of the Protective Effects of Aqueous Bark Extract of Terminalia arjuna (Roxb.) on Isoproterenol-Induced Cardiac Hypertrophy in Rats
Source: Front Pharmacol. 2019 Dec 10;10:1443. doi: 10.3389/fphar.2019.01443 (PMC6916006; doi:10.3389/fphar.2019.01443)

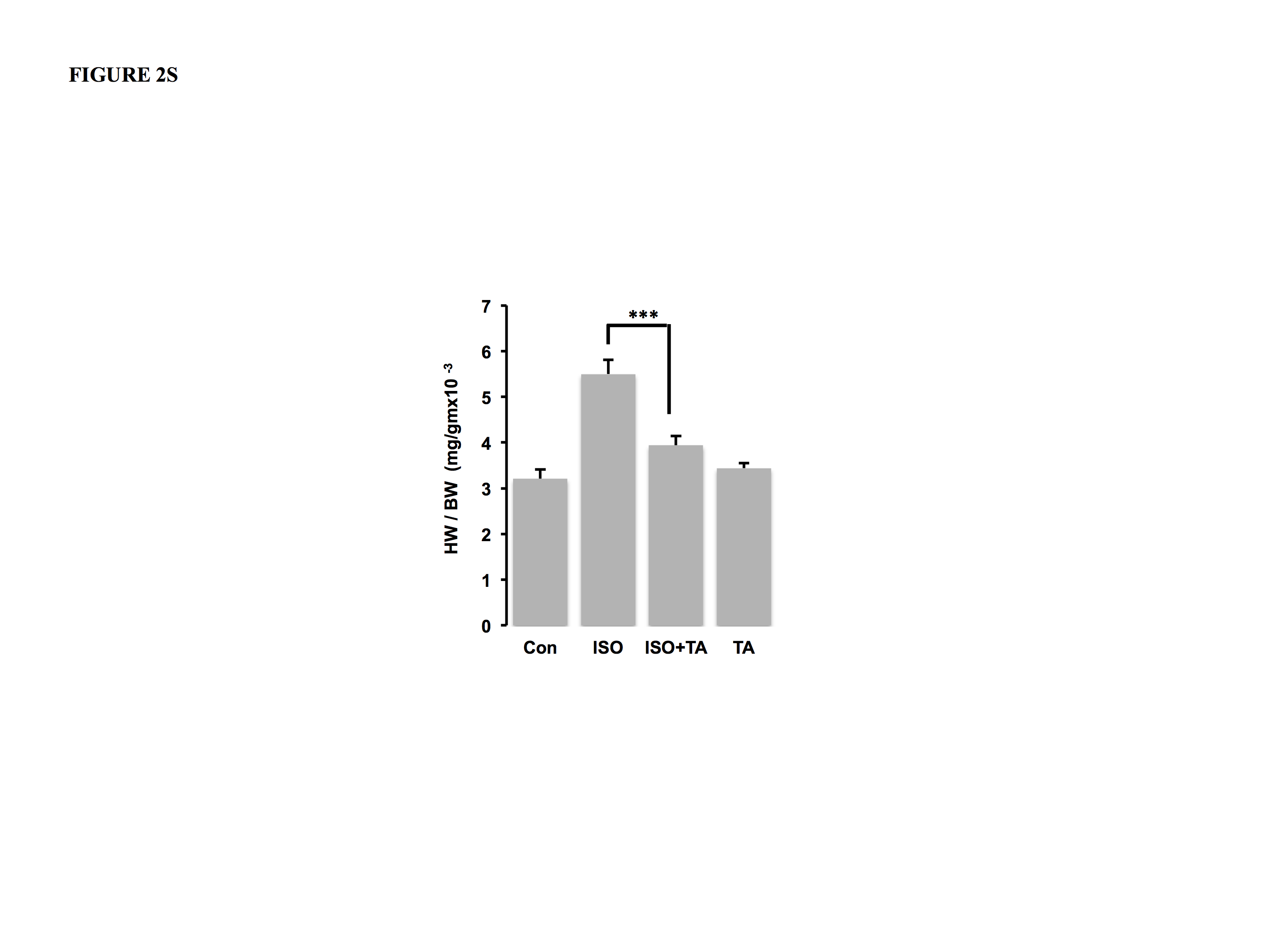

Supplement: Supplementary file 2 [file Image_1.tiff]
